# Evolution and plasticity of morph‐specific integration in the bull‐headed dung beetle *Onthophagus taurus*

**DOI:** 10.1002/ece3.6711

**Published:** 2020-09-02

**Authors:** Patrick T. Rohner, Anna L. M. Macagno, Armin P. Moczek

**Affiliations:** ^1^ Department of Biology Indiana University Bloomington IN USA

**Keywords:** geometric morphometrics, Onthophagini, phenotypic plasticity, Scarabaeidae, secondary sexual trait compensation

## Abstract

Developmental and evolutionary processes underlying phenotypic variation frequently target several traits simultaneously, thereby causing covariation, or integration, among phenotypes. While phenotypic integration can be neutral, correlational selection can drive adaptive covariation. Especially, the evolution and development of exaggerated secondary sexual traits may require the adjustment of other traits that support, compensate for, or otherwise function in a concerted manner. Although phenotypic integration is ubiquitous, the interplay between genetic, developmental, and ecological conditions in shaping integration and its evolution remains poorly understood. Here, we study the evolution and plasticity of trait integration in the bull‐headed dung beetle *Onthophagus taurus* which is characterized by the polyphenic expression of horned (‘major’) and hornless (‘minor’) male morphs. By comparing populations subject to divergent intensities of mate competition, we tested whether mating system shifts affect integration of traits predicted to function in a morph‐specific manner. We focussed on fore and hind tibia morphology as these appendages are used to stabilize major males during fights, and on wings, as they are thought to contribute to morph‐based differences in dispersal behavior. We found phenotypic integration between fore and hind tibia length and horn length that was stronger in major males, suggesting phenotypic plasticity in integration and potentially secondary sexual trait compensation. Similarly, we observed that fore tibia *shape* was also integrated with relative horn length. However, although we found population differentiation in wing and tibia shape and allometry, populations did not differ in integration. Lastly, we detected little evidence for morph differences in integration in either tibia or wing shape, although wing allometries differed between morphs. This contrasts with previous studies documenting intraspecific differentiation in morphology, behavior, and allometry as a response to varying levels of mate competition across *O. taurus* populations. We discuss how sexual selection may shape morph‐specific integration, compensation, and allometry across populations.

## Graphical abstract

We sought to further our understanding of how population divergence in the strength of sexual selection shapes morph‐specific trait integration and secondary sexual trait compensation across populations in a polyphenic dung beetle. We find evidence for integration between tibia shape and size with horn length, but little divergence among populations with diverging mating systems. We discuss how the strength of sexual selection shapes morph‐specific trait integration, secondary sexual trait compensation, and allometry across populations.

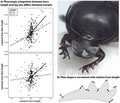

## 1 INTRODUCTION

Organisms can be viewed as mosaics of traits that covary with each other to varying degrees. Such covariation, or integration, represents a hallmark of biological systems and reflects both an evolved property and one that has the potential to influence subsequent evolution by constraining or biasing selectable phenotypic variation (Armbruster, Pélabon, Bolstad, & Hansen, 2014; Badyaev, 2010; Cheverud, 1996; Falconer, ; Hulsey, Hollingsworth, & Holzman, 2010; Murren, 2012; Olson & Miller, 1999; Schluter, 1996; Simmons & Garcia‐Gonzalez, 2011). Understanding the origin and evolution of phenotypic trait covariation, as well as its effect on population divergence, is thus integral to our understanding of how and why populations and species diverge the way they do.

Phenotypic integration can be adaptive if natural selection favors the co‐occurrence of several traits via correlational selection or selection on certain functional trait combinations on the evolutionary, static, and ontogenetic levels (Cheverud, 1982, 1984; Klingenberg, 2014). Examples include evolutionary integration of ecological variation, life history, physiology, and behavior within and among species (Blanckenhorn et al., 2020; Réale et al., 2010; Ricklefs & Wikelski, 2002), developmental integration between morphology and behavior among individuals (Beckers, Kijimoto, & Moczek, 2017), or the ontogenetic integration between functionally related skeletal structures (Zelditch, 1988). In addition to such adaptive scenarios, trait covariation can also arise as a by‐product of shared developmental processes involved in the development of different traits, or arise neutrally by mere genetic correlation via pleiotropy or linkage (Lande, 1982; Lynch & Walsh, 1998).

The evolutionary causes and consequences of integrated syndromes are best understood on the species and population level (e.g. anoles (Losos, Jackman, Larson, de Queiroz, & Rodríguez‐Schettino, 1998), cichlids (Kocher, Conroy, McKaye, & Stauffer, 1993; Montaña & Winemiller, 2013), and sticklebacks (Rundle, Nagel, Boughman, & Schluter, 2000)), yet similar ecological differentiation can also be found *within* populations, most notably in species with polyphenic development related to alternative mating or survival tactics. Polyphenic development enables the same genotype to generate discrete morphs as a function of environmental conditions, often yielding drastic intraspecific divergence in life history, behavior, and morphology in the process (e.g., microbivorous and omnivorous morphs in nematodes (Ragsdale, Müller, Rödelsperger, & Sommer, 2013) and amphibians (Ledón‐Rettig & Pfennig, 2011), dispersal and life cycle polyphenisms in insects (Brisson, 2010; Tauber et al., 1986), predator‐induced polyphenisms in *Daphnia* (Tollrian & Dodson, 1999) and rotifers (Stemberger & Gilbert, 1984), castes in social insects (Miura, 2005), and heterophylly in plants (Schmalhausen, 1949; Wells & Pigliucci, 2000)). As the divergent ecologies of different morphs are likely to favor alternate trait combinations and covariation within morphs, selection is expected to shape the evolution of morph‐specific patterns of integration. This may be especially pronounced when alternate morphs relate to alternate mating tactics as the strength of sexual selection commonly exceeds that of natural selection (Andersson, 1994; Hosken & House, 2011). Furthermore, mating polyphenisms are frequently accompanied by the evolution of exaggerated secondary sexual weaponry (e.g. head and thoracic horns in beetles (Moczek & Emlen, 2000), chelicerae in harvestmen (Painting, Probert, Townsend, & Holwell, 2015), or mandibles of longhorn beetles (Goldsmith, 1985)), whose morph‐specific development may necessitate highly divergent morph‐specific patterns of integration with other supporting or compensatory structures (Husak & Swallow, 2011), a phenomenon referred to as secondary sexual trait compensation (Tomkins, Kotiaho, & LeBas, 2005).

Here, we investigate the evolution and plasticity of phenotypic integration in the polyphenic dung beetle *Onthophagus taurus* (Schreber, 1759). Male *O. taurus* exhibit tightly choreographed, plastic changes in morphology, life history, physiology, and behavior as a response to resource availability experienced during larval development (Moczek, 2003). Male larvae with access to abundant food develop into large adults yielding a pair of large, curved head horns used as weapons in aggressive male–male combat over mating opportunities. In contrast, male larvae with limited access to larval nutrition emerge at a smaller adult size and develop minute horns. These “minor” males engage in nonaggressive sneaking behaviors and are more frequently subject to postcopulatory selection (such as sperm competition), rather than precopulatory male–male combat (Buzatto, Tomkins, & Simmons, 2014). The scaling relationship between horn size and body size is strongly sigmoidal, with a critical threshold size separating small, hornless minor males from large, fully horned, “major” males. Even though “intermediate” male morphologies do exist in natural populations, their rarity contributed to the two male morphs of *O. taurus* originally being described as separate species (Paulian, 1935).

Morphological differences among morphs are not restricted to the possession of horns, however, and also include differences in the size of the fore tibia and the hind wing, respectively. The fore tibia is a shovel‐like enlarged leg region endowed with large tibial teeth and functions in digging (Linz, Hu, & Moczek, 2019). In the context of male combat, however, it also serves to stabilize fighting males within tunnels as they exchange head butts with their opponent (Moczek & Emlen, 2000). Tomkins et al. (2005) described a positive relationship between relative horn length and relative fore tibia size in major males (i.e. the ‘fighting morph’) and attributed this to secondary sexual trait compensation—an adaptive form of trait integration between secondary sexual traits and other structures that act in concert with the sexually selected trait or compensate for or ameliorate the fitness costs that trait exaggeration brings about. Put another way, males that yield disproportionately large horns for their body size also develop disproportionately longer fore legs as supporting structures. Because relative tibia size increased more strongly with relative horn length in major compared with minor males, Tomkins et al. (2005) suggested this to be a case of phenotypically plastic trait compensation. Hind wings, in contrast, do not function directly in the context of fights but play a critical role in adult dispersal. Hunt, Kotiaho, and Tomkins (1999) documented that major *O. taurus* disperse more readily than their minor male counterparts and develop relatively larger wings. Hind wings therefore, too, exhibit morph‐specific patterns of trait variation. However, how such morph‐specific patterns of integration arise and may diversify in response to divergent selective regimes remains unclear. In particular, the interplay between genetic, developmental, and ecological conditions in shaping trait integration and its evolution remains poorly understood (Esteve‐Altava, 2017). Taking advantage of recent introductions of *O. taurus* to non‐native environments, we sought to investigate the evolution and plasticity of morphological integration across populations subject to divergent levels of mate and resource competition.

*Onthophagus taurus* is native to the Mediterranean, including Italy and Spain, but became introduced—among other places—to Western Australia (WA) (Bornemissza, 1976) and the Eastern United States (EUS) (Hoebeke & Beucke, 1997). Upon introduction, exotic populations underwent differential climatic niche expansion (DaSilva, Vilela, Buzatto, Moczek, & Hortal, 2016) alongside heritable divergences in life history, behavior, and secondary sexual trait expression (Beckers, Anderson, & Moczek, 2015; Casasa & Moczek, 2015; Macagno, Beckers, & Moczek, 2015; Macagno, Moczek, & Pizzo, 2016; Moczek & Nijhout, 2002; Parzer, David Polly, & Moczek, 2018; Rohner & Moczek, 2020). EUS populations are generally characterized by low densities, low levels of male competition for females, and female competition for nesting opportunities. In contrast, Western Australian populations are generally characterized by high densities often two orders of magnitude above those seen in the EUS range, intense male competition for females, and severe competition among females (from both conspecifics and other co‐occurring and highly abundant *Onthophagus* species) for nesting opportunities (Moczek, 2003). Population‐specific variation in density, resource availability, and operational sex ratios strongly suggest that EUS and WA populations are subject to diverging mating systems (Emlen & Oring, 1977; Kokko, Klug, & Jennions, 2014). Moreover, many of the heritable trait differences thus far documented for these exotic populations are consistent with adaptive differentiation in traits indicative of specific mating system components. Most importantly, WA and EUS populations have diverged heritably in the threshold body size separating alternate male morphs and morph ratios, resulting in a higher body size threshold and relatively fewer horned males in WA, consistent with predictions derived from status‐dependent selection theory (Moczek, 2003; Moczek & Nijhout, 2003). However, whether corresponding population divergences also exist for morph‐specific wing or tibial size and/or shape is thus far unknown.

In this study, we first quantified population differentiation in male fore tibia and wing shape, and allometry across two native as well as two exotic populations with divergent levels of male–male and resource competition. We then investigated morph‐specific patterns of phenotypic integration between relative horn length and relative tibia and wing size and shape across these same populations. We predict stronger integration between horns, tibiae, and wings in major males compared with minor males regardless of population because tibiae and horns function collectively in the execution of fights, and past work indicates that horned males disperse more effectively by flight. Further, we expect morph‐specific integration for tibiae and horns to diverge among populations due to the likely increased strength of sexual selection acting on the horned morph in WA population, or to the relatively higher frequency of the horned morph in the EUS population, or both. More generally, we sought to further our understanding of how population divergence in the strength of sexual selection shapes morph‐specific phenotypic integration and secondary sexual trait compensation across populations.

## 2 MATERIALS AND METHODS

### 2.1 Beetle husbandry and morphometric measurements

**FIGURE 1:**
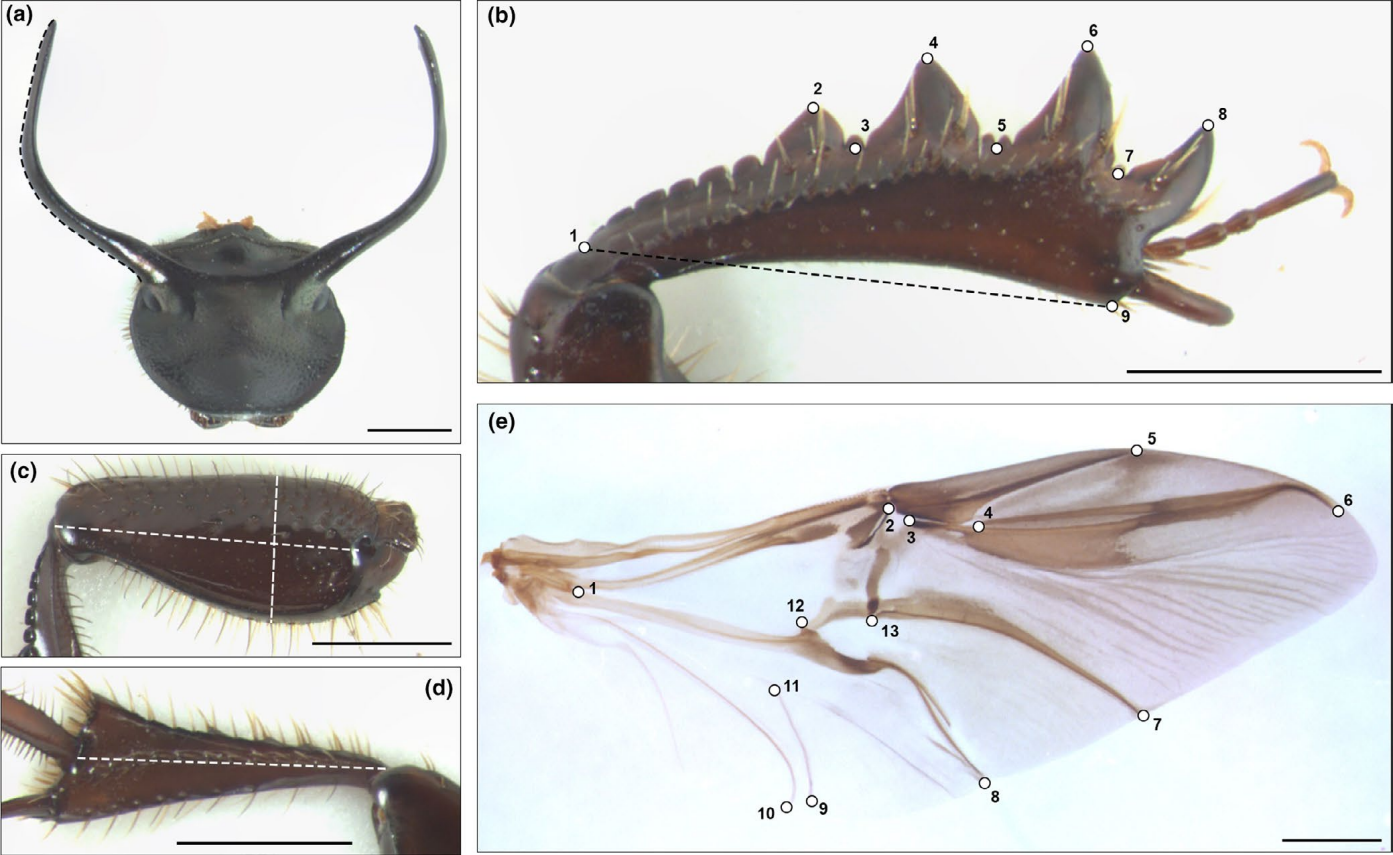
Morphometric measurements taken for all individuals (*n* = 224 total). To quantify horn length (a), we followed Moczek (2003) (see Moczek, 2006). To quantify fore tibia shape, we used 9 two‐dimensional landmarks. The distance between landmarks 1 and 9 was used to measure fore tibia length. Fore femur width and length (c) and hind tibia length (d) were measured by ordinary distance measures as indicated in the plots. Hind wing shape was quantified by placing 13 two‐dimensional landmarks on prominent wing vein positions (e). Scale bar, 1 mm

Adult beetles were collected from two invasive populations in Western Australia (WA) (Busselton, Western Australia) and the Eastern United States (EUS) (Chapel Hill, North Carolina), as well as in the native range in Italy (IT) (Monte Cucco, Umbria) and Spain (SP) (Seville, Andalusia). Wild‐caught individuals were shipped to Bloomington, Indiana, to be reared in laboratory colonies under standard laboratory conditions (see e.g. Macagno et al., 2016). Because fore tibia morphology is subject to significant wear when used for digging, tibia morphology of wild individuals is not well suited to study variation in shape. We therefore used the first filial generation of field‐caught individuals to quantify static allometry and integration of fore tibia shape and size. Male F1 offspring of Spanish (*n* = 59), Italian (*n* = 49), Western Australian (*n* = 55), and North Carolinian females (*n* = 61) were killed shortly after adult emergence and complete hardening to prevent digging and tibial wear. Care was taken to sample individuals that cover the full range of body sizes and horn morphologies within populations. Specimens were stored in 70% EtOH until dissected for morphometric measurements. We dissected and photographed the fore tibia and fore femur, the hind tibia, the head horns, and the pronotum using a digital camera (Scion) mounted on a Leica MZ‐16 stereomicroscope. TpsDig2 (Rohlf, 2009) was then used to measure fore tibia length, fore femur length and width, hind tibia length, horn length (following Moczek, 2006), and pronotum width (see Figure 1). Fore tibia shape was described using 9 two‐dimensional landmarks as described in Figure 1, and centroid size was used as a shape‐independent measure of overall structural size (Klingenberg, 2016).

The left hind wing was dissected and mounted on a glass slide using glycerol. The use of glycerol allowed us to apply pressure onto the coverslip, thereby fully flattening wings while obtaining images. Wing shape and centroid size were quantified using 13 homologous landmarks marking distinct wing venation features (Figure 1).

Morph identity was assigned based on horn length. Males with horns longer than 2.7 mm were considered majors, while all other specimens were considered minors (note that this also includes “intermediates,” but because minors and intermediates show similar horn shape allometries (Crabtree, Macagno, Moczek, Rohner, & Hu, 2020), they were pooled in this analysis).

### 2.2 Population differentiation in shape and allometry

We used a Procrustes ANOVA (using the function *procD.lm()* as implemented in *geomorph* (Adams & Otárola‐Castillo, 2013)) to test for population and morph differences in allometry in wing and tibia shape using type III Sums of Squares and removing nonsignificant interaction terms. To illustrate the major axes of population differentiation in wing and tibia shape, we further used a canonical variate analysis (CVA) with Jackknife cross‐validation (as implemented in the R‐package *Morpho* (Schlager, 2017)). This technique finds the linear combinations of shape variables (canonical variates) that differentiate best between group means (Klingenberg, Duttke, Whelan, & Kim, 2012; McCune, Grace, & Urban, 2002; Zelditch, Swiderski, & Sheets, 2012).

Because the Procrustes ANOVA indicated that wing shape allometries differed between morphs and populations (significant log centroid size × population and log centroid size × morph − interactions; see Section 3), morph‐specific multivariate regressions of shape on size were calculated for each population separately. The vectors of coefficients of these regressions represent the multivariate “broad‐sense” form of static allometry for each morph per population, respectively (Klingenberg, 2016). These vectors were then used to compute allometric spaces using ordination of allometric vectors to illustrate variation in allometric scaling (Gerber, Eble, & Neige, 2008; Gerber & Hopkins, 2011; Rohner, 2020; Strelin, Benitez‐Vieyra, Fornoni, Klingenberg, & Cocucci, 2018). To this end, we used the R‐function *prcomp()* based on the covariance matrix of all static allometric vectors. Such ordination resulted in an allometric space where each point represents an allometric vector (rather than an individuals' shape as in an ordinary morphospace), where distances between points relate to the similarity in this particular allometric space.

### 2.3 Morphological integration with horn length

**FIGURE 2:**
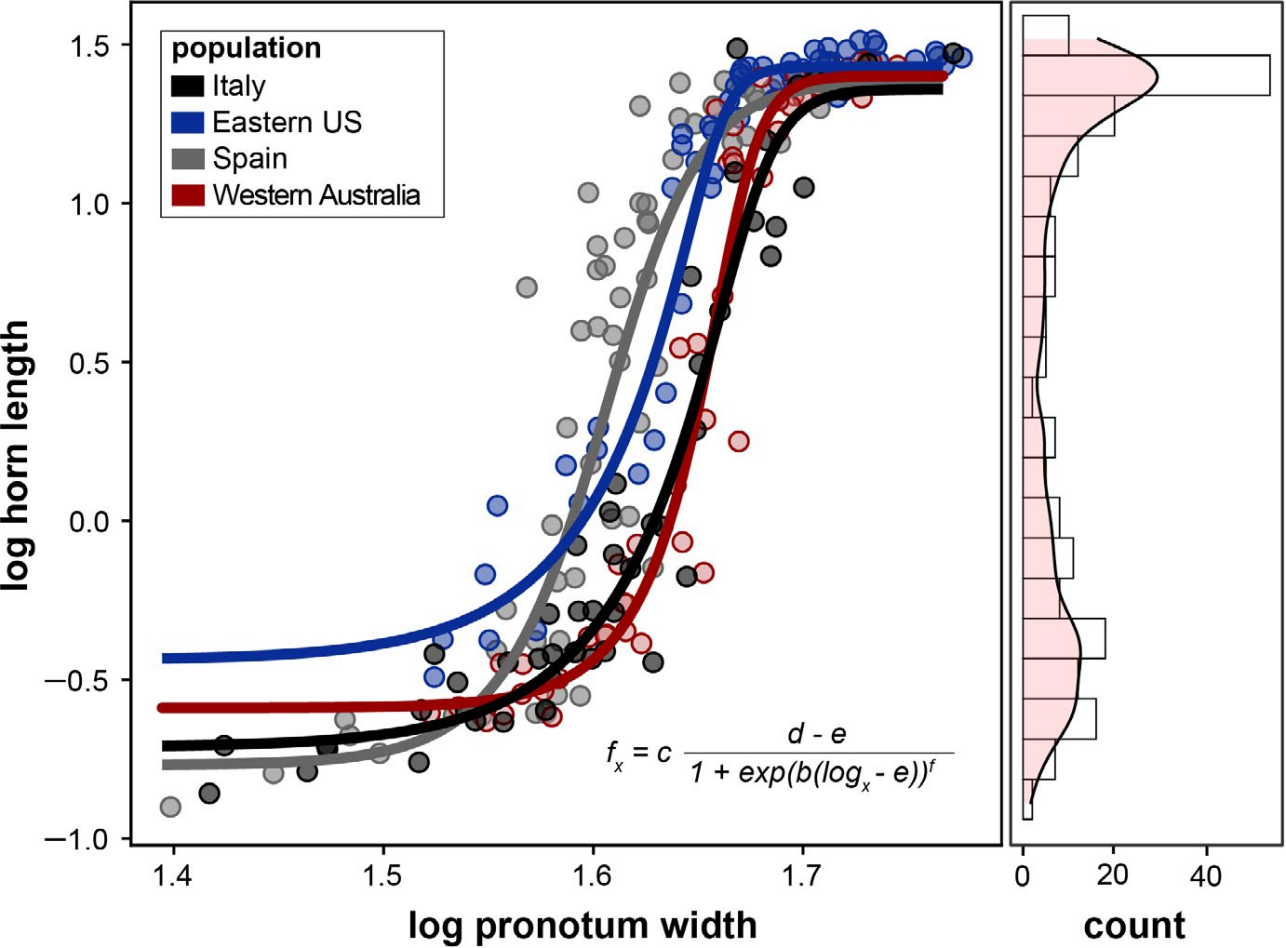
The scaling relationship between log horn length and body size (log pronotum width) is strongly sigmoidal and divergent among populations. The histogram to the right indicates a bimodal distribution of horn length. Given that we purposely sampled males to cover the full range of body sizes and horn morphologies within populations with divergent horn allometries, individuals with intermediate horn morphologies are overrepresented. The bimodal distribution found here is therefore much weaker compared with what is found in natural populations (see, e.g., Moczek & Emlen, 1999)

Because horn length shows a strongly sigmoidal scaling relationship in this species (Moczek, 2003; Moczek & Nijhout, 2003), we used a 5‐parameter log‐logistic regression to fit the allometric relationship of log horn length with log pronotum width (a widely used measure of body size in the Onthophagini c.f. Emlen (1994)) using the R‐package *drc* (Ritz, Baty, Streibig, & Gerhard, 2016; see Figure 2). Because populations differ in their scaling relationship, separate sigmoid models were fitted per population (thereby lowering the AIC from 152.4 to 22.0; Figure 2).

To assess phenotypic integration with horn length, we tested for a relationship between relative horn length and the relative size and shape of all other traits. To this end, we extracted residual trait size from linear regressions of log fore tibia length, fore tibia centroid size, fore femur length and width, hind tibia length, and wing centroid size against log pronotum width for each population separately. We then used a linear model to test for a relationship between these residual trait values and residual horn length as derived from the 5‐parameter log‐logistic regression, including interactions with morph and populations. Nonsignificant interaction terms were removed.

To test for covariation between relative horn length and wing and tibia shape, we again first removed body size variation by using multivariate regressions of tibia and wing shape against log centroid size for each population separately. We then used a Procrustes ANOVA (with type III Sums of Squares) to test for a relationship between residual wing and tibia shape and relative horn length including population, morph, and all interactions. Nonsignificant interaction terms were removed. Repeating the same analyses with two‐block partial least squares analysis (PLS) rendered qualitatively similar results (data not shown). All statistical analyses were conducted in R (R Core Team, 2016), unless stated otherwise.

## 3 RESULTS

### 3.1 Population differentiation in shape

We sought to better understand the evolution of morph‐specific phenotypic integration in response to divergent ecological circumstances in a microevolutionary framework. Despite the short time in allopatry among the native and two exotic *O. taurus* populations studied here, we found population differentiation in wing and tibia shape, consistent with rapid differentiation paralleling morphological as well as life history, physiological, and behavioral traits reported previously (Macagno et al., 2015; Moczek, 2003). For fore tibiae, the dominant canonical variate (CV1; explaining 57.6% of the total variance) mainly related to the length of the tibial teeth and mostly discriminated between the North Carolinian and the Italian and Western Australian population, with Spanish individuals somewhere in between. CV2 (26.9%) on the other hand related to a relative broadening of the tibia and mainly distinguished Spanish from all the other populations. Overall, the CVA for tibiae had a cross‐validated classification success of 73.4%. The four populations can therefore be identified well based on their fore tibia morphology.

**FIGURE 3:**
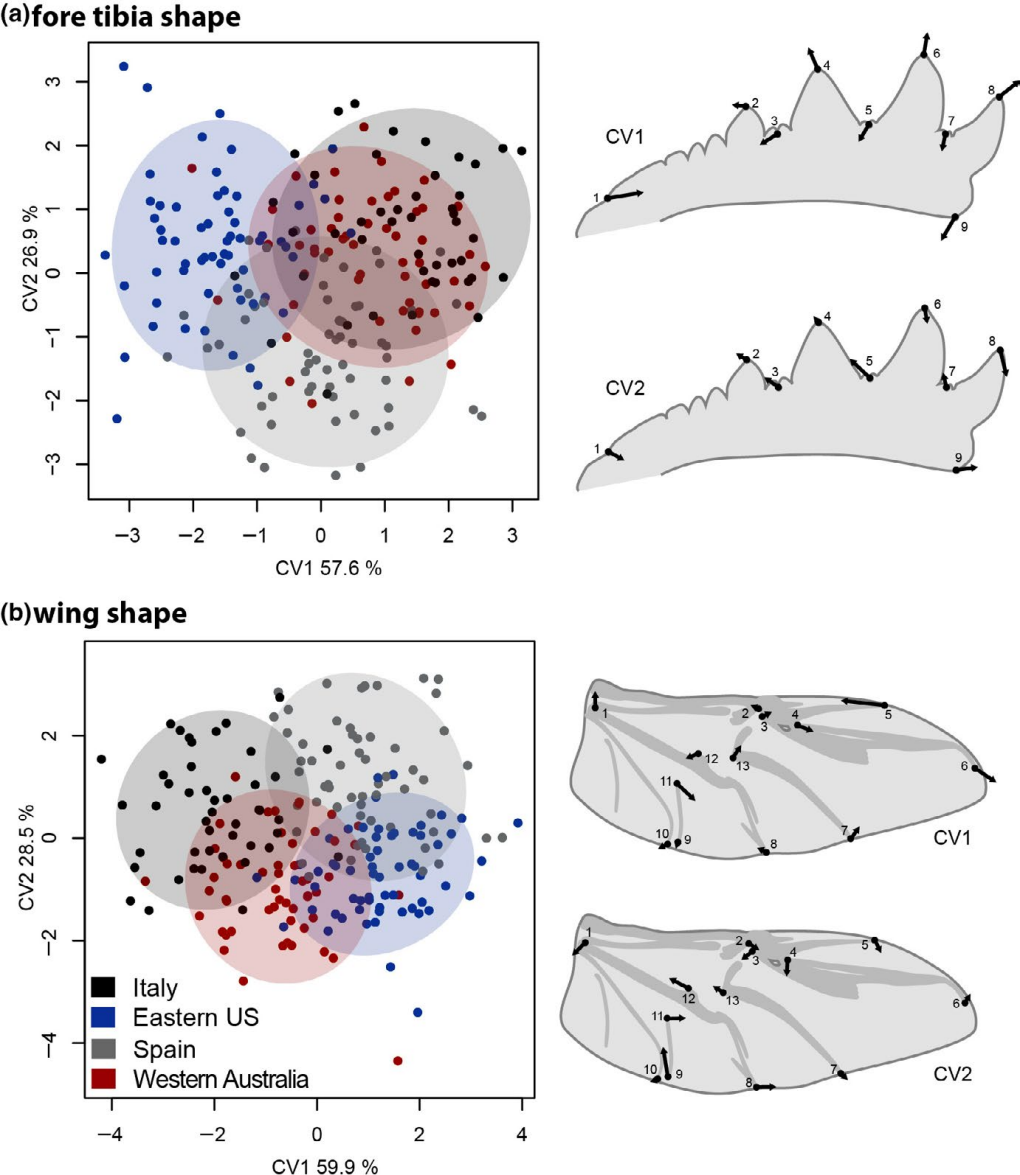
Canonical variate analysis (CVA) of tibia and wing shape reveals the main axes of population differentiation. Tibia shape differed between populations, although there was a pronounced overlap between Italian and Australian individuals in the morphospace spanned by the first two canonical variates (a). Populations also differed in wing shape. Australian and Italian individuals again cluster together on CV1. Shaded areas indicate 95% confidence ellipses

Similar patterns were recovered for hind wing morphology. CV1 (59.9%) describes mainly differences in the positioning of landmarks 5 and 6 at the tip of the wing and differentiates between Italian and Western Australian versus Spanish and Eastern US beetles (see Figure 3b). CV2 (28.5%) mainly differentiates the ancestral European from the two invasive populations. The cross‐validation success amounted to 76.9% for wings. When controlling for body size, the loadings as well as the cross‐validation success of the CVAs remain very similar (not shown), indicating that differentiation in body size and allometry play a minor role in overall population differentiation in tibia and wing shape. Both tibia and wing shape therefore differentiate rather well between the four populations investigated here, whether population differences in allometry are accounted for or not.

### 3.2 Population differentiation in allometry

**FIGURE 4:**
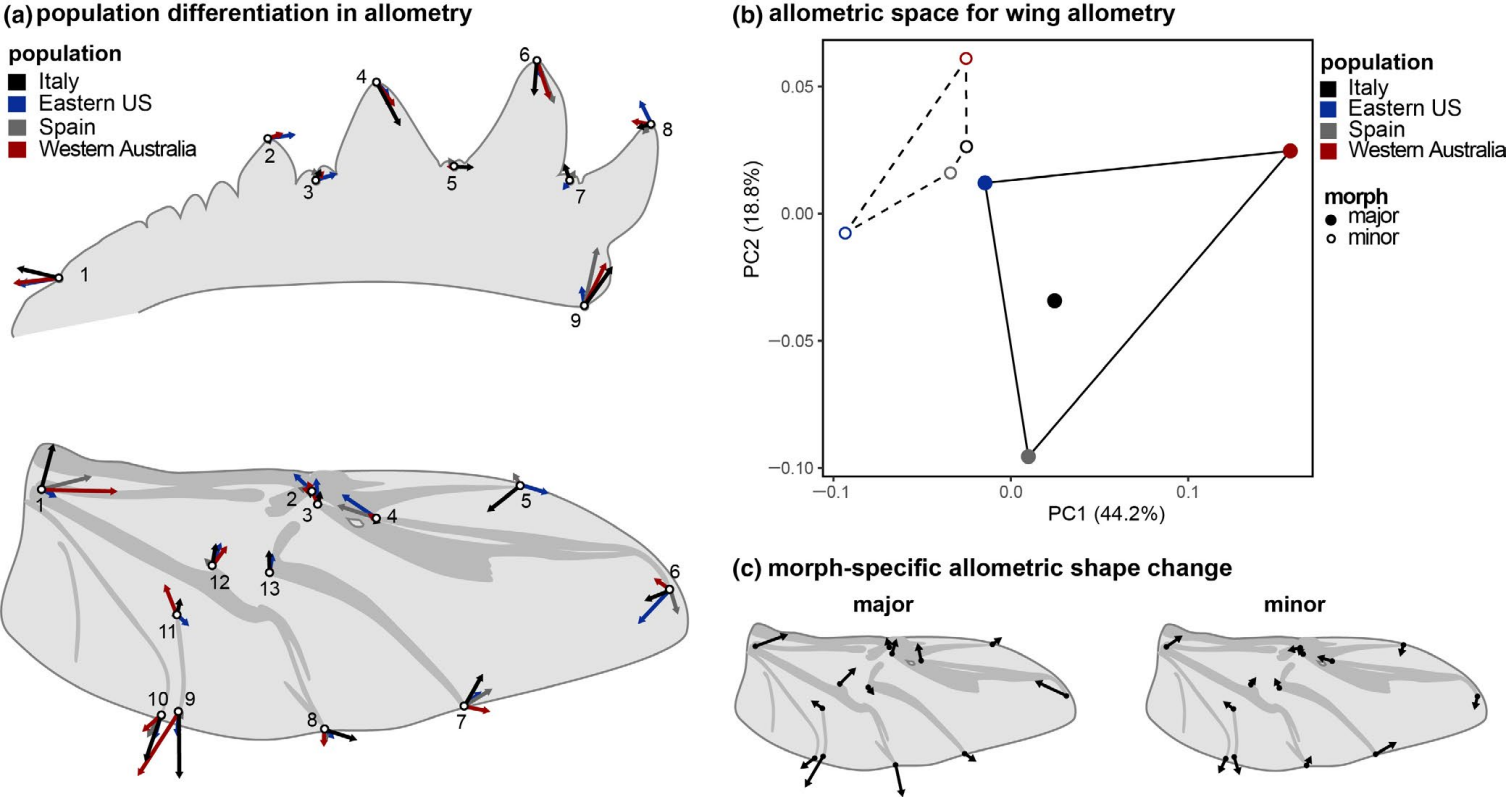
Population differentiation and morph‐specific static allometric relationships for tibia and wing morphology. (a) Larger individuals have longer and more slender fore tibiae with less pronounced tibial teeth. Allometry in wing morphology mostly related to broader and more roundish wings with increasing size. (b) Ordination of the static allometric relationships for wings in an allometric space indicated an additional effect of male morph on wing allometry in that the broadening of the wing was much stronger in major males compared with minor males (c)

Larger individuals have longer and more slender fore tibiae with less pronounced tibial teeth (Table 2a, Figure 4a). This relationship was generally similar in all populations but was, nevertheless, statistically different between them. Allometry in wing morphology mostly related to broader and more roundish wings with increasing size (i.e. lower aspect ratio with increasing size; Table 2a, Figure 4a). This relationship differed between populations. In addition to the general effect of size, the Procrustes ANOVA as well as ordination of the static allometric relationships in an allometric space (Figure 4b) indicated an additional effect of male morph on wing allometry in that the broadening of the wing was much stronger in major males compared with minor males (Figure 4c, Table 2a). This suggests that populations diverged in the allometric scaling in tibia shape as well as wing shape, with the latter also showing morph‐specific patterns.

### 3.3 Morphological integration with horn length

**FIGURE 5:**
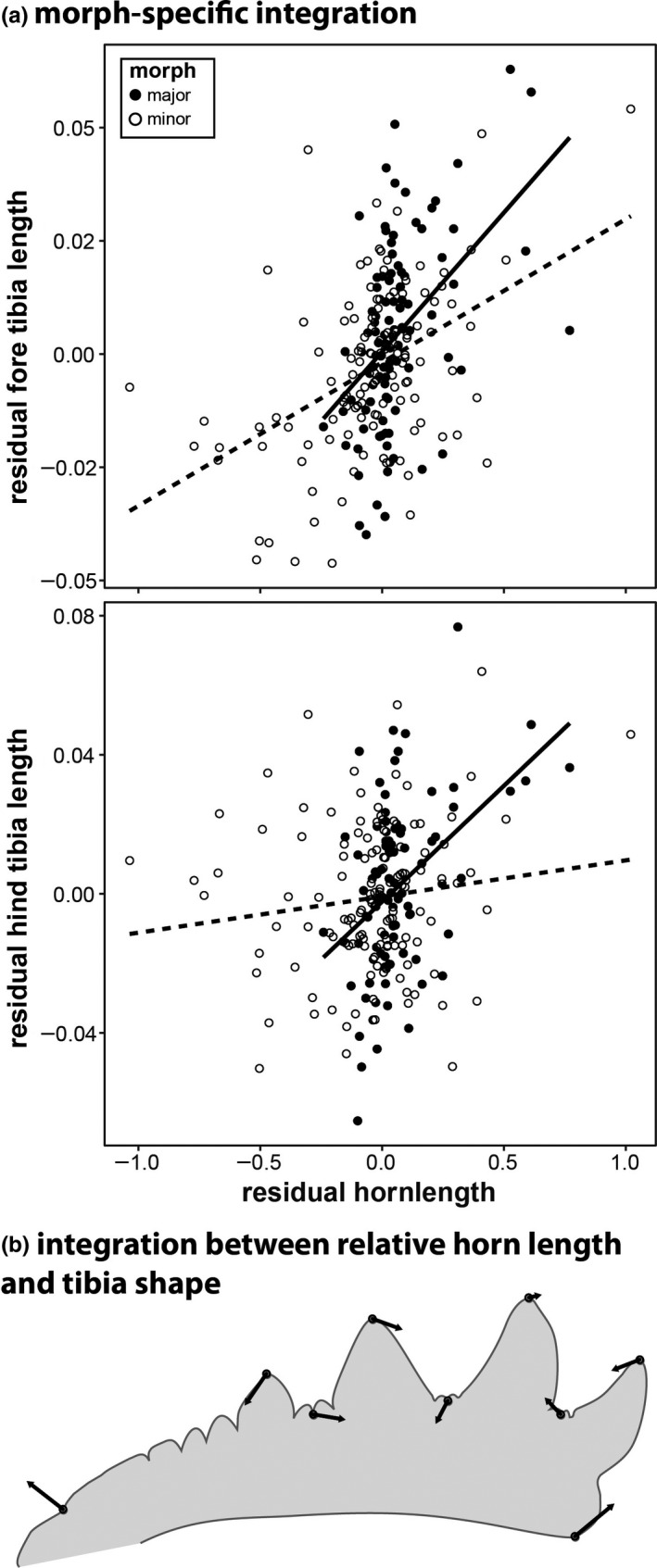
(a) morph‐specific patterns of integration for fore and hind tibia length. (b) changes in tibia morphology associated with an increase in relative horn length

Males with disproportionately long horns had larger appendages in general, as evidenced by a positive effect of relative horn length on all traits measured (Table 1). This relationship depended on morph for the length of fore and hind tibiae, where major males showed a stronger increase in relative trait size with relative horn size (significant interaction term, Table 1; Figure 5a). This might indicate a developmental reprogramming of the scaling relationship between relative horn length and leg length that leads to sexual trait compensation.

**TABLE 1:** Integration between relative (i.e., residual) trait size and relative horn size was tested using ANOVAs

	(a) Residual fore tibia length				(b) Residual tibia centroid size			
	SS	*df*	*F*	*p*	SS	*df*	*F*	*p*
Intercept	6.40 × 10^5^	1	0.20	.652	2.40 × 10^4^	1	0.98	.325
Residual horn length	8.59 × 10^3^	1	27.50	<.001	7.74 × 10^3^	1	31.42	<.001
Population	4.00 × 10^5^	3	0.04	.988	1.13 × 10^4^	3	0.15	.928
Morph	3.01 × 10^4^	1	0.96	.327	5.62 × 10^4^	1	2.28	.132
Residual horn length × morph	1.58 × 10^3^	1	5.04	.026				
Residuals	6.59 × 10^2^	211			5.23 × 10^2^	212		

	(c) Residual fore femur length				(d) Residual fore femur width			
Intercept	8.30 × 10^5^	1	0.22	.637	4.40 × 10^5^	1	0.10	.750
Residual horn length	9.59 × 10^3^	1	25.78	<.001	4.66 × 10^3^	1	10.83	.001
Population	3.90 × 10^5^	3	0.04	.991	2.10 × 10^5^	3	0.02	.997
Morph	1.87 × 10^4^	1	0.50	.479	1.15 × 10^4^	1	0.27	.606
Residuals	7.88 × 10^2^	212			9.13 × 10^2^	212		

	(e) Residual hind tibia length				(f) Residual wing centroid size			
Intercept	2.61 × 10^4^	1	0.55	.461	6.80 × 10^5^	1	0.37	.543
Residual horn length	1.08 × 10^2^	1	22.67	<.001	6.58 × 10^3^	1	35.97	<.001
Population	1.70 × 10^4^	3	0.12	.949	3.20 × 10^5^	3	0.06	.981
Morph	2.15 × 10^4^	1	0.45	.504	1.78 × 10^4^	1	0.97	.325
Residual horn length × morph	6.17 × 10^3^	1	12.90	<.001				
Residuals	1.01 × 10^1^	211			3.88 × 10^2^	212		

Note

Nonsignificant interaction terms were removed. All traits covary positively with relative horn length, although this relationship was stronger in major compared with minor males for relative fore and hind tibia length.

In contrast to fore tibia length, integration with fore tibia centroid size was not dependent on morph, indicating differences in tibia shape, rather than overall size. Correspondingly, we also found significant associations between relative horn length and residual tibia shape, that is, those animals with disproportionately large horns also possessed more slender fore tibiae (Table 2, Figure 5b). However, in contrast to tibia length, this relationship did not differ between morphs or populations (Table 2). We also did not find any significant integration between horn length and wing shape. Population‐by‐morph interactions were nonsignificant throughout and were thus removed. Combined, these findings suggest that despite integration between horn length and tibia shape, and in contrast to allometry, there is little evidence for population or morph divergence in the integration for wing and tibia shape.

**TABLE 2:** (a) Size‐dependent variation, that is, allometry, in fore tibia and wing shape was tested using Procrustes ANOVAs including population and male morph. (b) Covariation between relative (i.e., residual) horn length and residual tibia and wing shape was tested using Procrustes ANOVAS (type III SS). In all models, nonsignificant interaction terms were removed

(a) Allometry	Fore tibia shape					Hind wing shape				
	SS	*df*	*F*	*Z*	*p*	SS	*df*	*F*	*Z*	*p*
Log centroid size	6.80 × 10^3^	1	11.62	5.11	<.001	9.75 × 10^4^	1	4.06	3.58	<.001
Population	5.94 × 10^3^	3	3.38	4.40	<.001	1.58 × 10^3^	3	2.18	3.43	<.001
Morph	1.53 × 10^3^	1	2.61	2.25	.011	5.53 × 10^4^	1	2.30	2.27	.009
Log centroid size × population	4.51 × 10^3^	3	2.57	3.51	<.001	1.55 × 10^3^	3	2.14	3.36	<.001
Log centroid size × morph						5.54 × 10^4^	1	2.30	2.28	.008
Residuals	1.24 × 10^1^	212				5.07 × 10^2^	211			

(b) Integration	Residual fore tibia shape					Residual hind wing shape				
Population	1.04 × 10^4^	3	0.06	−9.68	1.000	2.60 × 10^5^	3	0.04	−12.20	1.000
Residual horn length	1.22 × 10^3^	1	2.12	1.78	.033	2.21 × 10^4^	1	0.93	0.06	.492
Morph	5.37 × 10^4^	1	0.94	0.10	.476	1.33 × 10^4^	1	0.56	−1.08	.859
Residuals	1.21 × 10^1^	212				5.04 × 10^2^	212			

## 4 DISCUSSION

We here sought to better understand the evolution and plasticity of integration across distinct morphs and populations. By comparing evolutionary lineages subject to divergent intensities of mate competition, we tested whether mating system shifts were accompanied by the evolution of phenotypic covariation. Our microevolutionary approach rendered three main results: First, we found phenotypic integration between fore and hind tibia length and horn length that was stronger in major males (i.e. the ‘fighter morph’). This corroborates previous findings and suggests phenotypic plasticity in integration possibly related to secondary sexual trait compensation. Second, fore tibia *shape* was integrated with relative horn length. However, although we found population differentiation in wing and tibia shape and allometry, populations did not differ in integration. Third, in contrast to fore tibia *length*, integration between horn length and tibia *shape* did not differ between morphs. Furthermore, while wing allometry differed between morphs, we did not find any evidence for morph‐specific integration in wing shape. This contrasts with previous studies that document strong intraspecific differentiation in morphology, behavior, and allometry as a response to mating system shifts in *O. taurus* (Casasa & Moczek, 2018; Macagno et al., 2015, 2016; Moczek, Hunt, Emlen, & Simmons, 2002).

### 4.1 Behavioral ecology and developmental plasticity of integration

Sexual selection frequently shifts secondary sexual traits from their viability optimum (Hosken & House, 2011). While these fitness costs are expected to be offset by increased reproductive success (Andersson, 1994; Blanckenhorn, 2007), they can additionally be ameliorated by the evolution of concerted variation in structures that either enhance the function of the secondary sexual trait or compensate for the inflicted fitness costs (Husak & Swallow, 2011; Møller, Lindén, Soler, Soler, & Moreno, 1995). This should then trigger the evolution of integration between secondary sexual traits and supporting or compensating structures. We here found that major males with disproportionately large horns also develop longer fore and hind tibiae, corroborating the results found by Tomkins et al. (2005). As both pairs of legs are used to brace males against tunnel walls during male–male contests (Moczek & Emlen, 2000), this positive covariation likely enhances a male's competitive ability and may, in addition, compensate for other fitness costs inflicted by large horns. For instance, horned males suffer impeded maneuverability due to horns scraping against tunnel walls (Moczek & Emlen, 2000; also see: Madewell & Moczek, 2006), and it is conceivable that changes in tibial morphology may compensate for costs inflicted by exaggerated horn morphology. However, whether the covariation found here is indeed adaptive and driven by correlational selection, or instead caused by mere linkage, neutral pleiotropy, or independent adaptation or variation, remains to be tested.

In addition to size, we also found integration between relative horn length and tibia shape. Tibia shape, in conjunction with size, might also contribute to fighting as the forelegs are braced against the tunnel wall during combat. Notwithstanding, in contrast to size, integration of shape was not morph‐specific, as would have been expected if mating tactics are the main driver of integration. However, the degree to which minors and majors differ in their fighting behavior may not be as discrete as oftentimes assumed. Polyphenic variation in mating strategies is frequently portrayed as highly polarized, with a ‘fighting’ morph showing male–male aggression and fighting behavior, whereas males that fall in the ‘sneaker’ category exclusively employ more clandestine tactics (Simpson, Sword, & Lo, 2011). Yet, in *O. taurus*, fighting behavior is not restricted to major males (Beckers et al., 2017; Moczek & Emlen, 2000). Specifically, if minor males encounter opponents of similar size, they will also engage in male–male combat identical in posture and motion to their horned male counterparts (Moczek & Emlen, 2000). Only if the opponent is considerably larger, individuals will abstain from aggressive interactions and instead engage in alternate sneaking behaviors. The propensity of escalating fights is therefore more dependent on the competitor's relative size, than on morph identity (although body size and trait exaggeration can be statistically, as well as biologically entwined, e.g. Baur et al., 2020; Gould, 1966). Especially in populations where majors are rare and densities are high, such as in Western Australia, minors may be more likely to engage in male–male combat rather frequently. If tibiae play a direct role in fighting, one may therefore not necessarily predict integration to differ between morphs but to rather scale with size. Nevertheless, the morph‐dependent integration in tibia size could relate to other aspects of polyphenic development as well. For example, during courtship males tap the female's elytra repeatedly with their forelegs (‘drumming’; see: Beckers et al., 2017; Kotiaho, 2002), and females exert preferences based on courtship vigor (Kotiaho, Simmons, & Tomkins, 2001; Simmons & Holley, 2011), therefore, tibia shape and integration may also function in the context of ornamentation for courtship, rather than weaponry for combat. Alternatively, or in addition, major males of Eastern US origin engage in significantly more paternal assistance during reproduction, including the digging of tunnels, than their minor male counterparts (Moczek, 1999), which could further shape the morph‐dependent integration in tibia size.

In contrast to tibia shape and size, there was no evidence for morph‐specific integration between relative horn size and wing shape (or size). However, we did find differences in wing shape allometry between morphs in that the wing aspect ratio decreases more strongly with size in majors compared with minors (Figure 4c). That is, large major males have disproportionately rounder and broader wings. As lower aspect ratios can increase maneuverability (Dudley, 2002), the morph‐specific allometric adjustments may relate to the ecological differences found between morphs in nature (Hunt et al., 1999). At the same time, individuals with disproportionately large horns also had relatively larger wings (as reported by Hunt et al. 1999), again suggesting trait compensation. However, this relationship did not differ between morphs, and the relationship beetween wing shape and its plasticity to variation in flight capacity remains poorly understood, especially in systems in which different morphs may be subject to different aerodynamic conditions and flight ecologies.

### 4.2 Population differentiation in shape and allometry

Exotic *O. taurus* populations are characterized by rapid evolution of life history, morphology, and behavior that are thought to be adaptive responses driven by among‐population differences in the strength of competition for mates and nesting resources (Macagno et al., 2015, 2016; Moczek, 2003). Correspondingly, we expected the major axes of tibia and wing shape differentiation to capture differences between native and exotic populations. Although we found population differentiation, the two European populations did not cluster together. Instead, Italian specimens grouped with the Australian population in horn allometry (Figure 1), tibia shape (CV1 and 2; Figure 2a), and wing shape (CV1; Figure 2b). This pattern hints at potential heterogeneity and population structure within the native range and highlights the need for a better population genetic understanding among native populations and their respective relationship to exotic lineages.

The allometric scaling of wings and tibiae differed between populations, yet the deformations associated with changes in body size were overall similar across populations in both direction and magnitude (Figure 4a). However, whether population differentiation in shape or allometry of any of the traits studied here are functionally significant, let alone reflective of adaptive divergences, remains to be established. Nevertheless, the patterns described here motivate future work as the relationship between geometric morphometric variation of size, shape, and fitness of complex traits is generally poorly understood (but see e.g.: Baur et al., 2020; Gomez & Perfectti, 2010).

### 4.3 Does mate competition drive the evolution of integration?

Although selection may mediate strength and form of integration (Gómez, Perfectti, & Klingenberg, 2014; Rosas‐Guerrero, Quesada, Armbruster, Perez‐Barrales, & Smith, 2011; Tomkins et al., 2005), we found no evidence for population differentiation in integration with horn length. We thus failed to detect support for our initial hypothesis that mating system shifts, either due to variation in operational sex ratios or mate and resource competition in exotic populations, may drive population differentiation in integration. Multiple reasons may be able to explain this result. For example, differences in selective regimes among populations might simply be too weak or too variable, or differentiation in integration may be evolutionary or developmentally constrained. However, it is worth pointing out that by studying how relative trait sizes covary with relative horn length, we studied integration on the level of the developmental system of an *individual* independent of size. Secondary sexual trait compensation could, however, also function via a *genotype's* static trait‐specific scaling relationships with size. Put another way, covariation between two traits may not be driven by a direct developmental or genetic relationship, but rather via covariation of each trait with overall body size (or any other intrinsic parameter). After all, in its broadest definition, allometry refers to any relationship or covariation between trait size and overall body size (Klingenberg, 2016), and because most traits show some kind of scaling relationship, most traits covary with each other. Allometry *sensu* *lato* thus causes strong covariation among the sizes of most traits and represents a considerable source of ontogenetic, static, and evolutionary integration (Klingenberg & Marugan‐Lobon, 2013; Olson & Miller, 1999). Secondary sexual trait compensation via shared covariation with size is probably the most common and most fitness relevant form of trait compensation. Population differentiation in allometries, or morph‐dependent scaling relationships, as found here, may therefore contribute to integration and compensation. Nevertheless, future work is needed to clarify to what extent allometries are driven by selection on function of a given trait in relation to body size, in relation to other traits (i.e. integration), or other evolutionary forces.

## 5 CONCLUSIONS

Phenotypic integration and secondary sexual trait compensation play a major role in the development and evolution of costly exaggerated weapons and ornaments and contribute to morph‐specific development in polyphenic species. We here corroborate previous studies suggesting morph‐specific and potentially adaptive forms of covariation between relative horn length and tibia length. However, such developmental plasticity in integration was not detected for tibia shape nor for wing size or shape. There was also little evidence that divergent ecologies and mating systems led to the evolution of trait integration. Yet, multivariate scaling relationships are, in and of themselves, forms of integration and may contribute to secondary sexual trait compensation in polyphenic species. Morph‐dependent developmental plasticity in scaling relationships may therefore represent a generally underestimated source of integration and secondary sexual trait compensation in polyphenic systems.

## CONFLICT OF INTERESTS

The authors have no conflict of interest to declare.

## AUTHOR CONTRIBUTION

**Patrick T. Rohner:** Conceptualization (lead); Data curation (lead); Formal analysis (lead); Funding acquisition (equal); Methodology (equal); Visualization (lead); Writing‐original draft (lead); Writing‐review & editing (equal). **Anna L. M. Macagno:** Conceptualization (supporting); Data curation (supporting); Methodology (supporting); Resources (equal); Supervision (supporting); Writing‐review & editing (supporting). **Armin P. Moczek:** Conceptualization (equal); Funding acquisition (equal); Resources (lead); Writing‐original draft (equal); Writing‐review & editing (equal).

## Data Availability

All data underlying this study are deposited on Dryad (https://doi.org/10.5061/dryad.1rn8pk0rh).
